# The most important medicinal plants affecting sperm and testosterone production: a systematic review

**DOI:** 10.5935/1518-0557.20210108

**Published:** 2022

**Authors:** Shakiba Nasiri Boroujeni, Farid Ansari Malamiri, Fatemeh Bossaghzadeh, Alireza Esmaeili, Emadoddin Moudi

**Affiliations:** 1 Medical Plants Research Center, Basic Health Sciences Institute, Shahrekord University of Medical Sciences, Shahrekord, Iran; 2 Faculty of Veterinary Medicine, Islamic Azad University of Shahrekord, Shahrekord, Iran; 3 Department of Biology, Dezful Branch, Islamic Azad University, Dezful, Iran and Student Research Committee, Dezful Branch, Islamic Azad University, Dezful, Iran; 4 Non-Communicable Disease Research Center, Ilam University of Medical Sciences, Ilam, Iran; 5 Associate Professor, Department of Urology, Cancer Research Center, Health Research Institute, Babol University of Medical Sciences, Babol, Iran

**Keywords:** sperm production, testosterone, herb, traditional medicine, testicles, Iran

## Abstract

**Objective:**

Infertility, defined as the inability to conceive after one year of intercourse without the use of contraception, affects 15% of couples. Many factors, such as genitourinary tract infections, endocrine disorders, immunological factors and drug-related injuries, affect the male reproductive system and cause infertility. Due to men's fear of infertility, it is very important to pay attention to medicinal plants that are effective in male fertility. Therefore, the aim of this study was to evaluate the medicinal plants that affect sperm and testosterone production in men.

**Methods:**

In this review, we used the following search terms, consisting of herbal medicine, traditional medicine, traditional therapies, sperm, testosterone, testicles and Iran were used to retrieve the relevant articles published in the journals indexed in the Information Sciences Institute, Science Direct, PubMed, Scopus, PubMed Central and Scientific Information Databases. We searched and used papers published between 2000 and 2020. Then, we analyzed the eligible papers. We collected and analyzed 35 papers from the databases. We selected only the articles about herbs that affect sperm and testosterone production.

**Results:**

Based on the results, herbs *Apium graveolens, Cinnamomum camphora, Cornus mas, Satureja khuzestanica, Withania somnifera, Fumaria parviflora, zingiber officinale, cinnamomum zeylanicum and Phoenix dactylifera* are used in the male reproductive system.

**Conclusions:**

Plants can probably be useful in increasing fertility due to their antioxidant power and low side effects.

## INTRODUCTION

Infertility, defined as the inability to conceive after one year of intercourse without the use of contraception, affects 15% of couples (Kim *et al*., 2013). About 30-50% of infertility is related to male infertility and 30-40% of the causes of male infertility are related to sperm disorders (Godmann *et al*., 2009). The most common cause of infertility in men is their inability to produce enough healthy, active, and highly motile sperm (Bastampoor *et al*., 2014; Oyeyemi *et al*., 2008). Lack of testicular development, diseases of the reproductive system, increased scrotal temperature, immunological problems, endocrine disorders, lifestyle choices, environmental and nutritional factors are considered as the main causes of male infertility having a negative effect on sperm parameters (Marbeen *et al*., 2005; Sharpe & Franks, 2002; Low *et al*., 2013; Singh & Jemal, 2017; Chyou *et al*., 1993; De Rosa *et al*., 1981). Many factors, such as genitourinary tract infections, endocrine disorders, immunological factors, and drug-related issues, affect the male reproductive system and cause infertility (Wang *et al*., 2018; Jiang *et al*., 2017; Kesari *et al*., 2018; Agarwal *et al*., 2018). Disorders affecting spermatogenesis, hormone regulation, oxidative stress, and regulation of spermatogenesis-related genes cause infertility (Zhou *et al*., 2019; Moghbelinejad *et al*., 2018; Zhang *et al*., 2020; Hu *et al*., 2006; Ma *et al*., 2015). Infertility can be due to excessive consumption of natural plant compounds (phytoestrogens). These compounds can affect the reproductive system and reduce fertility (Csupor-Löffler *et al*., 2009). Sperm plasma membranes are exposed to oxidative damage due to large amounts of unsaturated fatty acids, which ultimately decrease sperm motility and viability. Antioxidant compounds increase sperm function and can improve fertility (Kooti *et al*., 2014).

According to several studies, oxidative stress can cause molecular and genetic defects causing infertility (Agarwal *et al*., 2006). Oxidative stress is usually associated with aerobic metabolism producing prooxidant molecules or reactive oxygen species (ROS). Some cells have specific mechanisms for producing the ROS required for cellular function at low concentrations. Depending on the tissue concentration of ROS, they can have beneficial physiological effects and play a role in the fertilization process. Free radicals can affect the ova, sperm, and embryos in their small environment. Free radicals can also damage cellular components, including lipids, proteins, and nucleic acids. There is a complex interaction of cytokines, hormones and other stressors that affect the production of free radicals (Gupta *et al*., 2007). ROS can be neutralized by a complex antioxidant defense system consisting of enzymes such as catalase, superoxide dismutase and glutathione peroxidase/reductase, and several non-enzymatic antioxidants such as vitamin C, vitamin E, vitamin A, pyruvate, glutathione, taurine (Safarnavadeh & Rastegarpanah, 2011). Whenever the level of ROS is pathologically elevated, antioxidants begin to work, helping to minimize oxidative damage, repair, or prevent it. The male and female reproductive organs are rich in both enzymatic and non-enzymatic antioxidants. Increased ROS levels can damage the ovum, the zygote/embryo, and most importantly, the sperm. Sperm are very sensitive to oxidative stress. Oxidative stress appears to be due to increased ROS production rather than a decrease in antioxidants (Safarnavadeh & Rastegarpanah, 2011). The WHO recommends the use of traditional medicines in the medical health care system. However, there has been a great deal of interest in finding natural antioxidants from herbal materials to replace synthetic drugs recently (Hasani-Ranjbar *et al*., 2009). There are many medicinal plants in the world with anti-fertility and fertility-enhancing properties (Jain *et al*., 2015; Modaresi *et al*., 2008). Many people now use herbs or their derivatives to increase or decrease fertility as well as libido (Kachroo & Agrawal, 2011). Some of these plants have spermicidal properties, others increase the number of sperm and change sperm motility. Some plants also alter testicular hormones (Khaki *et al*., 2014).

The World Health Organization reports that despite the increasing use of herbal medicines, there is still a significant lack of research on it, and the role of studies examining herbal medicines is crucial. Due to the clear negative effects of chemical drugs on humans, the tendency to use herbal medicines is increasing among women and men. We need to study the use of biologically active plant materials in the field of male fertility and to identify natural plant materials with estrogenic and anti-estrogenic properties (Marbeen *et al*., 2005). Due to men's fear of infertility (Hosseini & Abdi, 2012), it is very important to pay attention to medicinal plants that affect male fertility. Therefore, the aim of this study was to evaluate the medicinal plants that affect sperm and testosterone production in men.

## MATERIALS AND METHODS

We used the following search terms: herbal medicine, traditional medicine, traditional therapies, sperm, testosterone, testicles and Iran, to retrieve the relevant papers published in the journals indexed in the Information Sciences Institute, Science Direct, PubMed, Scopus, PubMed Central and Scientific Information Databases. Papers published in 2000-2020 were searched and used in the review. Then, we analyzed the findings of the eligible papers.

We assessed the abstracts for the pre-determined inclusion and exclusion criteria. We collected 35 papers from the databases, and 15 papers were taken out due to repeatability, irrelevance, lack of a summary, non-native Iranian plants, invalid papers and invalid journals. Papers without an abstract in English, without a full text and books were excluded from the analysis. Only the papers about herbs that affect sperm and testosterone hormone production were selected for the study (Flowchart 1).

**Flowchart 1:**
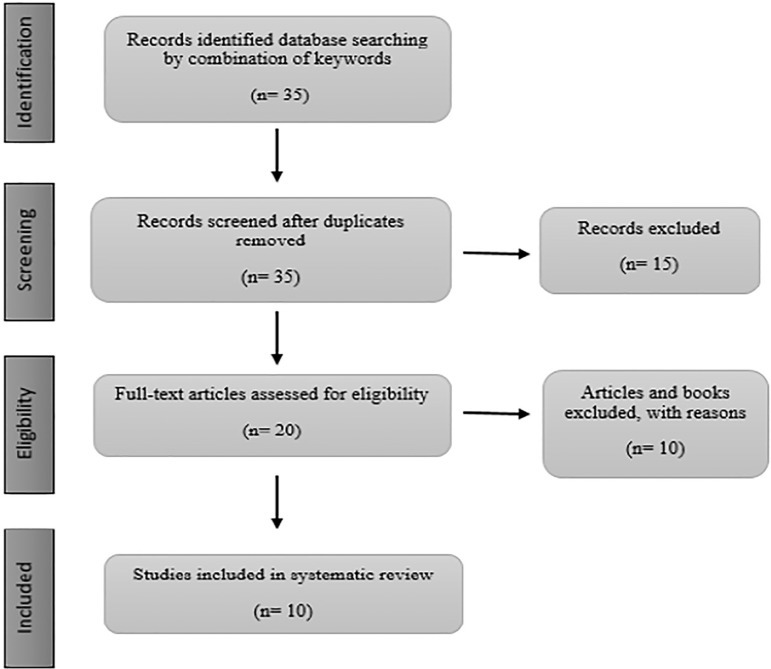
The criteria and the number of included and excluded articles.

## RESULTS

According to the analysis, 9 medicinal plants were found to affect the male reproductive system. Table 1 enlists some effective medicinal plants, their families, used organs, participants, intervention protocol, and the important findings. Also, the active ingredients of these herbs plus their chemical and molecular formula are listed in Table 2.

**Table 1.** The list of medicinal plants affects male reproductive system.

Plant	Family Name	Plant/extract	Participant	Intervention protocol	Result	Ref.
*Apium graveolens*	Apiaceae	Hydro- alcoholic Extract of leaves	Male Wistar rats	100 and 200 mg/kg/BW once every two days for 60 days, Orally	There was a significant increase in the number of sperms, sertoli cells, and primary spermatocyte in groups receiving extract; however, structural changes were not observed in the groups. It seems that celery increases spermatogenesis in male rats but has no destructive effects on testicular tissue. Perhaps this plant could be used to treat infertility in men.	Kooti *et al*., 2014
*Cinnamomum camphora*	Lauraceae	The active ingredient is prepared from plant stem	Male Wistar rats	1, 2 and 5 mg/Kg for 30 days, Intraperitoneally injection	Pure camphor in alcohol 10% increases LH level and decreases FSH level, whereas it failed to change level of testosterone. The claim of inhibitory effect of camphor on sexual activity could not be confirmed by this study.	Shahabi *et al*., 2014
*Cornus mas*	Cornaceae	Hydro-ethanolic extract of fruit	Male NMRI mice	250, 500 and 1000 mg/kg, Oral gavage	It was revealed that *Cornus mas* fruit extract decreased the cellular atrophy by controlling the energy substrate utilization based on lipids and carbohydrates via provoking the testicular antioxidant status. *Cornelian cherry* fruit extract, as an antioxidant compound, could reduce cellular degeneration, lower inflammation and up-regulate testicular antioxidant status. *Cornelian cherry* fruit extract plays a role in decreasing oxidative stress by increasing the TAOC. It can be concluded that this extract could protect reproductive organs against MTX side effects.	Zarei & Shahrooz, 2019; Zarei *et al*., 2014
*Satureja khuzestanica*	Lamiaceae	Essential oil of aerial parts	Male Wistar and Albino rats	75, 150, 225 mg/kg/day for 45 days and 225 mg/kg/day for 28 days, Orally	Significant improvements in potency, fecundity, fertility index, and litter size and significant decrease in post implantation loss. *Satureja khuzestanica* essential oil protected reproductive system from toxicity of Cyclophosphamide through its antioxidant potential and androgenic activity.	Safarnavadeh & Rastegarpanah, 2011 Haeri *et al*., 2006 Rezvanfar *et al.*, 2008
*Withania somnifera*	Solanaceae	Hydro-alcoholic and aqueous extract and powder of all parts mostly root	Human, cell, mice and rat	Different doses and most intervention protocol	It deems that *Withania somnifera* has a positive effect in the treatment of infertility both in male and female. Although some studies proposed that WS extract might have non-fertilizing and spermicidal effect.	Nasimi Doost Azgomi *et al*., 2018
*Fumaria parviflora*	Papaveraceae	ethanolic extract of leaves and powder	Male Wistar rats	200mg/kg day1 gavage for 70 days	The results indicated that ethanolic extract of *F. parviflora* leaves has potential to restore the suppressed reproduction associated with lead exposure and prevented lead-induced testicular toxicity in male Wistar rats.	Dorostghoal *et al.*, 2014
*zingiber officinale*	zingiberaceae	Ginger roots powdered and dissolved in 2cc distilled water	Male Wistar rats	100 mg/kg, gavage method, daily for, 8 weeks	The application of ginger plus Cinnamon compared with ginger and cinnamon alone in diabetic rats significantly improved the damaging effects of oxidative stress on spermatogenesis and fertility parameters. It seems that the antioxidant content of herbs could be increased dramatically when used in combination.	Khaki *et al*., 2014
*cinnamomum zeylanicum*	lauraceae	*Cinnamon zeylanicum *powdered and dissolved in 2cc distilled water	Male Wistar rats	75 mg/kg, gavage method, daily for, 8 weeks	The application of ginger plus Cinnamon compared with ginger and cinnamon alone in diabetic rats significantly improved the damaging effects of oxidative stress on spermatogenesis and fertility parameters. It seems that the antioxidant content of herbs could be increased dramatically when used in combination.	Khaki *et al*., 2014
*Phoenix dactylifera*	Arecaceae	extract of fruit	Human, rat, mice, rabbit, and hamster	Different doses in different routes	This review showed that *phoenix dactylifera* pollen is a very suitable supplement for infertility and can reduce free radicals and increase sperm motility.	Abdi *et al*., 2017 Fallahi *et al*., 2015
*Plukenetia conophora*	Euphorbiaceae	methanolic extract	Male Wistar rats	Different doses	Olaniyan et al. In 2018 to evaluate the effects of Plukenetia conophora (PC) and 4H-Pyran-4-One 2,3-Dihydro-3,5-Dihydroxy-6-Methyl (DDMP) on Wistar rats with chloride-induced testicular damage Cadmium (CdCl2). Treated daily for 54 days. Methanolic extract of Nigeria fruit seeds was used. Thus: control group (normal saline). CdCl2 (2 mg/kg single dose IP); CdCl2 + 200 mg/kg vitamin E; CdCl2 + 100 or 200 mg / kg PC; And CdCl2 + 25 or 50 mg / kg DDMP. The biochemical parameters of malondialdehyde, nitric oxide, antioxidant enzymes and proton pumps were measured by spectrophotometry. Reproductive hormones were measured using ELISA. In the treated groups, a significant increase in sperm count, motility and viability was observed. Malondialdehyde and nitric oxide levels were significantly reduced. Significant increases in antioxidant enzymes, proton pump and testosterone were observed in the treated groups.	Olaniyan *et al.*, 2018
*Garcinia kola*	Clusiaceae	ethanolic extract	Adult male Wistar rats	Different doses	It has been concluded that *Garcinia cola* and vitamin E show liver protection against oxygen free radicals produced by lead ions by maintaining the tissue integrity of rat testis.	Dare *et al*., 2014
*Cocos nucifera L*	Arecaceae	Oil	Adult male Wistar rats	Different doses	*Cocos nucifera* oil reduces the harmful effects of lead acetate in male Wistar rats, which may be due to its polyphenol content and antioxidant properties.	Dare *et al.*, 2012
*Adansonia digitata*	Malvaceae	aqueous extract	Male Wistar rats	Different doses	The cadmium chloride-treated group plus *A. digitata* caused a significant decrease in MDA levels with a significant increase in antioxidant activity and biochemical enzymes. The aqueous extract of A. digitata seems to have a healing effect against testicular damage caused by cadmium chloride. This can be attributed to the presence of a polyphenolic compound.	Olaniyan *et al*., 2021

**Table 2.** Herbal plants and chemical and molecular formula.

Scientific Name	The Most Bioactive Compound	Chemical Formula	Molecular Structure
*Apium graveolens*	Apigenin	C_15_H_10_O_5_	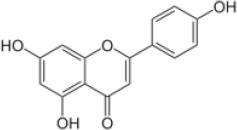
*Cinnamomum camphora*	Camphor (2-bornanon)	C_10_H_16_O	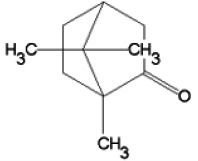
*Cornus mas*	Pelargonidin (Olaniyan *et al*., 2018)	Pelargonidin (Olaniyan *et al*., 2018)	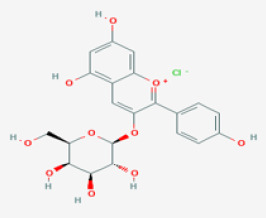
*Satureja khuzestanica*	Carvacrol (Dare *et al*., 2014)	C_10_H_14_O	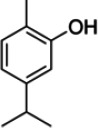
*Withania somnifera*	withaferin A	C_28_H_38_O_6_	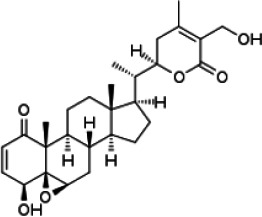
*Fumaria parviflora*	Protopine (Dare *et al*., 2012)	C_20_H_19_NO_5_	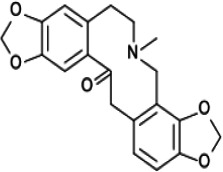
*zingiber officinale*	Gingerol (Olaniyan *et al*., 2021)	C_17_H_26_O_4_	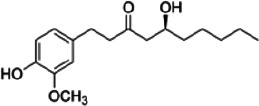
*cinnamomum zeylanicum*	Cinnamaldehyde (Dare *et al*., 2021)	C_9_H_8_O	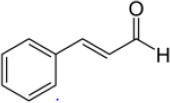
*Phoenix dactylifera*	Oleic acid (Bentrad *et al.*, 2017)	C_18_H_34_O_2_	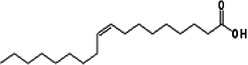

## DISCUSSION

Having children is one of the concerns of every couple after marriage, when it does not happen, it causes concerns for the couple and their families. Due to the support of the World Health Organization for maintaining public health and reproductive health, today the use of medicinal plants as a substitute or supplement to synthetic drugs affecting fertility is considered. This is a systematic study of Iranian medicinal plants effective in the treatment of male infertility.

Recent studies have shown that, under physiological conditions, reactive oxygen species play a very important role in the intracellular messaging processes. On the other hand, over the past decade, reactive oxygen species have been implicated in the development of male infertility due to their overproduction or a reduced ability of the reproductive system and sperm to deal with it. In pathological conditions, reactive oxygen species cause male infertility through impaired spermatogenesis, sperm function and structure, motility, viability, acrosome reaction, sperm-ovum mating, and even reduced fertilization and implantation (Dare *et al*., 2021; Bentrad *et al*., 2017; Fanaei *et al*., 2013).

There is a growing demand for herbal medicines around the world. Studies have shown that some medicinal plants may have fertility-enhancing properties in men by improving antioxidant activity, prevent the formation of free radicals and lipid peroxidation, and reduce oxidative stress, preventing damage to sperm cells (Moher *et al*., 2009). They also increase the number of testicular vessels, the lifespan and number of sperm, increasing sperm quality and protecting germ cells. On the other hand, these plants can enhance the activity of the hypothalamic-pituitary-gonadal axis on different levels, affecting the secretion of LH and testosterone (Oi *et al*., 2001; Ralebona *et al*., 2012). These medicinal plants affect male fertility, parameters such as sperm survival and mortality, pituitary hormone levels, histological changes in the testes and sperm depletion. Therefore, these herbs can help improve sperm parameters in infertile men, but this requires further clinical studies.

Some herbs inhibit the uptake of 5 alpha-reductase (a factor that converts testosterone into dihydrotestosterone), reducing gonadotropins and testosterone, increasing the affinity for sex-specific proteins, thickening the basement membrane, reducing germinal epithelial cells and reducing the irregular placement of these cells, reducing sperm count, motility, and sperm viability, but causing side effects such as infertility, at certain doses (Roozbeh *et al*., 2016). Some plants, such as garlic, inhibit the enzyme caspase 3 and cytochrome P450 2E1 (CYP2E1), which have a toxic effect on the testes, reducing testicular function and improving spermatogenesis by reducing these two enzymes (Vickers, 2017). *Garcinia cola* polyphenolic sections showed prophylactic effects on the histology and hormones of the pituitary-testicular axis of male Wistar rats (Omotola *et al*., 2017). Another study from Nigeria found that an injection of *Cissus populnea* root into male Wistar rats increased the secretion of male sex hormones such as testosterone and gonadotropins, thereby increasing the fertility of these rats (Olaolu *et al*., 2018). Similarly, *Cocos nucifera* water improved reproductive indices in Wistar rats (Kunle-Alabi *et al*., 2014). A study conducted in Iraq showed that *Cyperus esculentus* has a protective effect on testicular and sperm abnormalities caused by lead acetate in Wistar rats (Al-Shaikh *et al*., 2013). The aqueous extract of *Cyperus esculentus*, administered to Wistar rats for nine weeks, increased testicular and epididymal weight, increased sperm count and motility. These studies show that the medicinal properties of these plants are related to polyphenolic components, especially flavonoids, which can neutralize free radicals - the source of oxidative stress (Methorst & Huyghe, 2014). Flavonoids in plant samples have antioxidant power and can optimize the function of the body's antioxidant system. Reactive oxygen species are produced in a chain of reactions that alter male reproductive cells quantitatively and qualitatively; antioxidants act by interrupting these chain reactions (Methorst & Huyghe, 2014). In Iranian herbal medicine, many medicinal plants are used to treat various disorders and diseases. One of the most important reasons for the effectiveness of medicinal plants is the presence of effective medicinal substances (Solati *et al*., 2021; Bahmani *et al*., 2020; Aidy *et al*., 2020; Karimi *et al*., 2019; Abbasi *et al*., 2016; 2020). The effect of medicinal plants on sperm production and testosterone is due to the presence of secondary medicinal compounds. According to researchers, methods for standardizing medicinal plants, quality control, information on safety and effectiveness are needed to properly understand the use of herbal medicines. Finally, we recommend more experimental and clinical studies using modern scientific principles and methods in this field.

## CONCLUSION

Plants can probably be useful in increasing fertility due to their antioxidant power and lower side effects. The use of medicinal plants with the property of enhancing male fertility can be used as a substitute or supplement to chemical drugs that affect male fertility. On the other hand, it is recommended that plants with fertility reduction properties be used less or not at all in men with infertility disorders.
